# Effectiveness of 2D radiographs in detecting CBCT-based incidental findings in orthodontic patients

**DOI:** 10.1038/s41598-021-88795-3

**Published:** 2021-04-29

**Authors:** Jin-Young Choi, Song Hee Oh, Seong-Hun Kim, Hyo-Won Ahn, Yoon-Goo Kang, Yong-Suk Choi, Yoon-Ah Kook, Gerald Nelson

**Affiliations:** 1 Department of Orthodontics, Graduate School, Kyung Hee University, Seoul, Korea; 2 Department of Oral and Maxillofacial Radiology, Graduate School, Kyung Hee University, Seoul, Korea; 3 Department of Orthodontics, Postgraduate School of Dentistry, The Catholic University of Korea, Seoul, Korea; 4 Division of Orthodontics, Department of Orofacial Science, University of California–San Francisco, San Francisco, CA USA

**Keywords:** Anatomy, Diseases, Health care, Medical research

## Abstract

Some craniofacial diseases or anatomical variations are found in radiographic images taken for other purposes. These incidental findings (IFs) can be detected in orthodontic patients, as various radiographs are required for orthodontic diagnosis. The radiographic data of 1020-orthodontic patients were interpreted to evaluate the rates of IFs in three-dimensional (3D) cone-beam-computed tomography (CBCT) with a large field of view (FOV) and investigate the effectiveness and accuracy of two-dimensional (2D) radiographs for detecting IFs compared to CBCT. Prevalence and accuracy in five areas was measured for sensitivity, specificity, positive predictive value (PPV) and negative predictive value (NPV). The accuracies of various 2D-radiograph were compared through a proportion test. A total of 709-cases (69.5%) of 1020-subjects showed one or more IFs in CBCT images. Nasal cavity was the most affected area. Based on the CBCT images as a gold standard, different accuracies of various 2D-radiographs were observed in each area of the findings. The highest accuracy was confirmed in soft tissue calcifications with comprehensive radiographs. For detecting nasal septum deviations, postero-anterior cephalograms were the most accurate 2D radiograph. In cases the IFs were not determined because of its ambiguity in 2D radiographs, considering them as an absence of findings increased the accuracy.

## Introduction

Customary diagnostic records for orthodontic treatment planning include dental casts, intraoral and extraoral photographs, clinical examinations, radiographic data such as panoramic radiographs, lateral and postero-anterior cephalograms, and periapical x-rays^1^. While the information from limited data such as dental casts provides only the idea of relative postero-anterior relationship of the jaws or relative occlusion, radiographic data are necessary for diagnosing, planning treatment modalities, and evaluating the treatment results in that they provide more fundamental information such as the status of the jaws in the craniofacial complex^2^. Since the development of the radiation system for the cephalograms by Broadbent in 1931^3^ and the establishment of cephalometric analysis by Downs in 1948^4^, two-dimensional (2D) radiographs are used widely in orthodontics. In contemporary orthodontics, three-dimensional (3D) radiographs such as cone beam computed tomography (CBCT) have been used more frequently than before^5^. CBCT images provide information about skeletal deformity^6^, dental asymmetries^7^, impacted teeth^8,9^, and the quality of the alveolar bone housing^10^, and airway spaces^11^. For quality diagnosis of these conditions with 2D radiology, additional specialized views must often be requested.

Craniofacial diseases or anatomical variations without any symptoms of discomfort or deformity may be detected unexpectedly during radiographic examination for other purposes, such as the diagnostic tools for orthodontic treatment or dental implants^12,13^. Incidental findings (IFs) are defined as any abnormal or pathological findings that are unrelated to the original purpose of taking radiographic images^14^. Some of them are found and diagnosed accurately by 3D images, and might be missed with 2D radiographs such as panoramic or cephalometric radiographs^15^.

There have been reports on the prevalence of craniofacial diseases or abnormal structures; however, they have usually been based on the diagnosis of accompanying symptoms^16,17^. In terms of asymptomatic abnormalities, previous studies show that the 3D CBCT could detect IFs in areas outside of interest in patients who visit dental clinics. However, most studies used a relatively small number of subjects, less than 1000 images, and the FOV of the CBCT was small^14,18^. When the FOV value is small, only a few parts of the craniofacial area are viewed for evaluation^19^.

As described above, 2D radiographs such as panoramic radiographs and cephalograms are routinely taken in orthodontic patients. Although 2D radiographs are not as revealing as 3D radiographs, they can detect abnormal structures or diseases. Some authors have shown that IFs were found in 6% to 43% of 2D radiographic images^20–22^, while the rate of IFs in 3D CBCT was from 24.6% to 94.3%^18^. However, the accuracy of 2D radiographs in finding lesions incidentally has not yet been confirmed. Comparison of the ability between 2D and 3D CBCT radiology to detect IFs would be valuable since most clinicians tend to use 2D radiographs instead of 3D CBCT, given their lower radiation doses.

Therefore, this study aimed to investigate the rates of IFs of craniofacial diseases or abnormal structures using a large number of 3D CBCT images that were taken for orthodontic diagnosis without other symptoms, and to compare the detecting accuracy of 2D radiographs to that of 3D CBCT images.

## Materials and methods

### Samples

For this retrospective study, CBCT images were obtained from sorted patients who visited the Department of Orthodontics at Kyung Hee University Dental Hospital for orthodontic treatment with biocreative strategy from January 2010 to July 2019. CBCT radiographs were taken for orthodontic diagnosis only. All the experimental protocol with informed consent from all participants and the legally authorized representatives/parents/guardian/next of kin (in case of minors) for study participation was approved by the institutional review board of Kyung Hee University Dental Hospital (IRB No. KH-DT19022). The authors confirm that all methods were carried out in accordance with relevant guidelines and regulations.

The inclusion criteria were as follows: Korean patients who underwent CBCT for orthodontic treatment without any craniofacial symptoms, and CBCT images with C-mode, regardless of whether they proceeded orthodontic treatment after diagnosis. C-mode covers the craniofacial area with large field of view (FOV). A panoramic radiograph, lateral cephalogram, and/or a postero-anterior cephalogram were taken concurrently with the CBCT, based on the clinicians’ decision. There was no age restriction in this study. The exclusion criteria were patients who visited the clinic for orthodontic treatment to manage previously diagnosed craniofacial diseases, CBCT images, except the C-mode, and the cases whose radiographs were taken with intervals of one month or more. If multiple CBCTs were taken for one patient during the orthodontic treatment, only the first images were selected and the subsequent images were excluded. A total of 1020 CBCT images from 1020 patients were included (Table 1). As we selected patients according to the presence of CBCT images, some of them do not have 2D radiographic images such as a panoramic radiograph, lateral cephalogram, or postero-anterior cephalogram. The definite number of each radiographic image is shown in Table 2.

**Table 1:** Characteristics of subject.

Total number of patients	1020
**Age**	
< 10 years	101
10–19 years	428
20–29 years	303
30–39 years	89
40–49 years	53
> 50 years	46
Mean age, years	21.7
**Gender**	
Male	400
Female	620

**Table 2:** Number of radiographic image sets.

Radiographic images	Number of image sets (n)
PANO	999
LAT	1002
PA	849
PANO + LAT + PA	840
CBCT	1020

*PANO* panoramic radiograph, *LAT* lateral cephalogram, *PA* postero-anterior cephalogram.

### Radiographic equipment

CBCT images were obtained on the Alphard-3030 (Asahi Roentgen, Kyoto, Japan). In the C-mode, the tube voltage was 80 kVp, tube current was 10 mA, and the scan time was 17 s. A large area of craniofacial structure could be focused with 20 × 17.9 cm of FOV and 0.39 mm^3^ of isotropic voxel size. Lateral and postero-anterior cephalograms were obtained with the CX-90SP (Asahi Roentgen, Kyoto, Japan), and panoramic radiographs were obtained with three different devices: The Cypher E (Asahi Roentgen, Kyoto, Japan), Planmeca Promax (Planmeca Oy, Helsinki, Finland), and Orthopantomograph OP100 (Instrumentarium Dental, Tuusula, Finland). All radiographic data were saved as DICOM (digital imaging and communications in medicine) files in a picture archiving and communication system (Infinit Tech, Seoul, Korea) at Kyung Hee University Dental Hospital.

### Evaluation of radiographs

The images were evaluated by two oral and maxillofacial radiologists (S.H.O. and Y.S.C.) with imaging software (ZeTTA PACS, TaeYoung Soft, Anyang, Korea, http://taeyoungsoft.com/product01.php). The information such as name, age, and gender was blinded.

**Figure 1:**
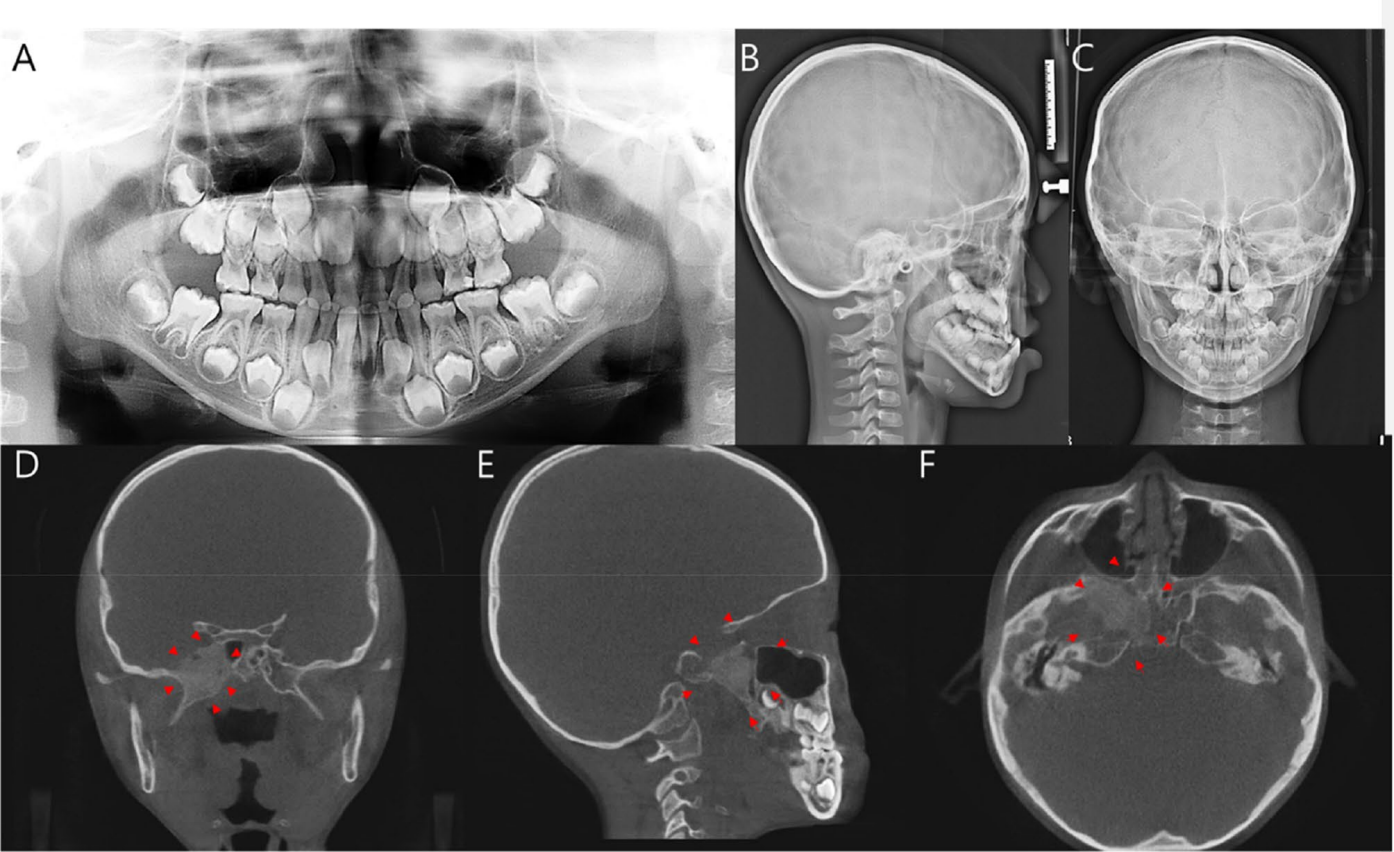
Fibrous dysplasia of the right sphenoid sinus was found on the 3D CBCT images of a 6-year-old male patient. The cortex had been thinned but was still intact, and granular internal bone pattern was observed on CBCT images, while no abnormal structures found on 2D radiographic images. (**A**) Panoramic radiograph, (**B**) lateral cephalogram, (**C**) postero-anterior cephalogram, (**D**–**F**) slices of CBCT showing abnormal bone pattern.

The IFs were classified into five categories: (1) maxillary sinus, (2) TMJ, (3) nasal cavity, (4) soft tissue calcification, and (5) pathology. In all five categories, CBCT readings were designated as gold standards based on the following: (1) In the maxillary sinus area, mucous retention pseudocyst, flat mucosal thickening (> 3 mm), polypoid mucosal thickening, partial opacification, and total opacification, based on a previous study (Fig. 1)^23^. (2) Any resorptive change in condyles in the IFs of the TMJ area. (3) Nasal septum deviation and concha bullosa for IFs of the nasal cavity, according to Mladina’s types^24^. A concha bullosa was defined as being present when more than 50% of the vertical height (measured from superior to inferior in the coronal plane) of the middle turbinate was pneumatized. 4) Clinically significant calcifications in the salivary glands, tonsils, or lymph nodes, and 5) any pathologic findings in the craniofacial area. The examples of pathologic findings included cystic change of bone, enlarged canals, and fibrous dysplasia, etc. All 2D radiographic images were evaluated by the same examiners, who evaluated CBCT images in the same manner. There were three types of 2D radiographs: panoramic radiograph, lateral cephalogram, and postero-anterior cephalogram. Some patients did not need all three radiographs for the orthodontic diagnosis. Therefore, the number of images were different depending on the type of radiographs (Table 2). To exclude the prejudgment of measurements by other images, the images of the same patients were not read concurrently but were merged for comprehensive evaluation.

Basically, all the 2D radiographic images were used to detect the IFs. However, there were a few exceptions due to the limitation of 2D images. Resorptive changes of the condyles could not be detected by lateral or postero-anterior cephalograms, and we were not able to find nasal septum deviation with lateral cephalograms. In addition, concha bullosa in the analysis with 2D radiographic images were excluded because it can be detected only in 3D images. When the findings were indefinite in 2D images because of the overlaps with other anatomic structures, the obscure border of lesions, and so on, they were classified as the “not determined” category.

### Statistical analysis

To evaluate the reliability of interpretation, 50 samples were selected randomly, and inter- and intra-examiner correlations were calculated with percent agreement and kappa statistics.

In each area, the data of 2D radiographic images that showed the highest accuracy were regarded as the reference, and the differences were calculated to determine the effectiveness of each 2D image in detecting IFs. The term “comprehensive radiographs” meant the decision was arrived at using all the 2D radiographs. If all three 2D radiographs had shown no IFs, we called it “absence” on the comprehensive radiographs. And if IFs had been detected on at least one radiograph, we considered it as “presence” on the comprehensive radiographs. In order to compare the distribution of readings in each set of 2D radiographic images to that in the 3D CBCT, kappa statistics was used. Cohen’s kappa coefficient provides the information about the similarity between two groups: < 0.2 means poor agreement; < 0.4 means fair agreement; < 0.6 means moderate agreement; < 0.8 means good agreement; and > 0.8 means very good agreement^25^.

As there was a number differences between the presence and absence of IFs in CBCT images, the sensitivity, specificity, positive predictive value (PPV), negative predictive value (NPV), and resultant accuracy could be distorted. Therefore, we selected cases randomly to match the number between the group in which IFs were detected and the group in which IFs were not detected with CBCT images. For example, as the number of presences of paranasal sinus findings on CBCT images was 151, 151 panoramic radiographs were supposed to be selected randomly. However, 6 of 151 patients do not have panoramic radiographs. In that case, we selected 145 panoramic radiographs randomly. On the other hand, 3 of 151 patients do not have lateral cephalograms, so 148 random lateral cephalograms were selected for the statistical analysis. Data in the “not determined” category were included to “absence” category for conservative interpretation. The cut-off value accuracy test was conducted with the following values:

$$Sensitivity={\frac{True \,\,Positive}{True\,\, Positive+False\,\, Negative}}\times 100$$

$$Specificity=\frac{True\,\, Negative}{True \,\,Negative+False \,\,Positive}\times 100$$

$$PPV=\frac{Sensitivity\times Prevalence}{Sensitivity\times Prevalence+(1-Speciviticy)\times (1-Prevalence) }\times 100$$

$$NPV=\frac{Specificity\times (1-Prevalence)}{Specificity\times (1-Prevalence)+(1-Sensitivity)\times Prevalence }\times 100$$

$$Accuracy=\frac{True \,\,Positive+True \,\,Negative}{True \,\,Positive+False \,\,Positive+True \,\,Negative+False\,\, Negative}\times 100$$

(PPV: Positive Predictive Value, NPV: Negative Predictive Value).

A proportion test was also conducted to compare the accuracy of each 2D radiograph and the statistical significance was evaluated with a P-value of 0.05. All statistical analyses were carried out with “caret” package of R 3.6.1 program (https://cran.r-project.org).

## Results

Intra- and inter-examiner agreements were over 80% in all variables. Kappa coefficients over 0.5 suggested that all the variables were in substantial agreement^26^.

### IFs detected on the CBCT images (Table 3)

**Table 3:** Incidental findings on CBCT images.

	Number of findings	Ratio of IFs, %
**Maxillary sinus**		
Mucous retention pseudocyst	36	3.53
Flat mucosal thickening (> 3 mm)	57	5.59
Polypoid mucosal thickening	35	3.43
Partial opacification	18	1.76
Total opacification	28	2.75
Total (number of subjects)	151	14.80
**TMJ**		
Resorptive changes	128	12.55
**Nasal cavity**		
Nasal septum deviation	602	59.02
Concha bullosa	251	24.61
Total (number of subjects)	676	66.27
Soft tissue calcification	10	0.98
Pathology	16	1.57

At least one IF was confirmed in overall 709 CBCT images, which accounted for 69.5% of all CBCT images. The most common IFs of all craniofacial areas were findings in the nasal cavity, especially nasal septum deviation. Ninety-three patients (9.1%) showed two or more IFs simultaneously. The mean age of the subjects with incidental findings was 22.3 years old, and the mean age of the subjects without incidental findings was 20.1 years old.

### IFs detected on the 2D radiographs (Tables 4 and 5)

**Table 4:** Prevalence of IFs by panoramic, cephalometric, and CBCT images.

	PANO	CBCT	Kappa	LAT	CBCT	Kappa	PA	CBCT	Kappa
**Maxillary sinus**									
Absence	819 (82.0%)	854 (85.5%)	0.434 (0.365–0.504)	975 (97.3%)	854 (85.2%)	0.191 (0.118–0.264)	764 (90.0%)	725 (85.4%)	0.306 (0.225–0.388)
Not determined	45 (4.5%)	0		7 (0.7%)	0		32 (3.8%)	0	
Presence	135 (13.5%)	145 (14.5%)		20 (2.0%)	148 (14.8%)		53 (6.2%)	124 (14.6%)	
**TMJ**									
Absence	874 (87.5%)	875 (87.6%)	0.453 (0.374–0.532)	N/A			N/A		
Not determined	27 (2.7%)	0		N/A			N/A		
Presence	98 (9.8%)	124 (12.4%)		N/A			N/A		
**Nasal cavity**									
Absence	486 (48.6%)	408 (40.8%)	0.180 (0.130–0.231)	N/A			387 (45.6%)	356 (41.9%)	0.320 (0.260–0.380)
Not determined	100 (10.0%)	0		N/A			32 (3.8%)	0	
Presence	413 (41.3%)	591 (59.2%)		N/A			430 (50.6%)	493 (58.1%)	
**Soft tissue calcification**									
Absence	994 (99.5%)	990 (99.1%)	0.641 (0.368–0.914)	998 (99.6%)	993 (99.1%)	0.613 (0.301–0.925)	848 (99.9%)	842 (99.2%)	0.248 (-0.147–0.644)
Not determined	1 (0.1%)	0		0	0		0	0	
Presence	4 (0.4%)	9 (0.9%)		4 (0.4%)	9 (0.9%)		1 (0.1%)	7 (0.8%)	
**Pathology**									
Absence	985 (98.6%)	982 (98.3%)	0.640 (0.468–0.812)	998 (99.6%)	985 (98.3%)	0.187 (0.003–0.37)	846 (99.6%)	835 (98.4%)	0.174 (0.030–0.318)
Not determined	4 (0.4%)	0		2 (0.2%)	0		3 (0.4%)	0	
Presence	10 (1.0%)	17 (1.7%)		2 (0.2%)	17 (1.7%)		0	14 (1.6%)	

*PANO* panoramic radiograph, *LAT* lateral cephalogram, *PA* postero-anterior cephalogram.

**Table 5:** Source of data in “not determined” category of each 2D radiograph.

		CBCT result	n	Ratio, %
Maxillary sinus	PANO	Presence	11	24.4
		Absence	34	75.6
	LAT	Presence	5	71.4
		Absence	2	28.6
	PA	Presence	10	31.3
		Absence	22	68.8
TMJ	PANO	Presence	6	22.2
		Absence	21	77.8
Nasal cavity	PANO	Presence	52	52.0
		Absence	48	48.0
	PA	Presence	12	37.5
		Absence	20	62.5
Soft tissue calcification	PANO	Presence	1	100
		Absence	0	0
Pathology	PANO	Presence	4	100
		Absence	0	0
	LAT	Presence	2	100
		Absence	0	0
	PA	Presence	3	100
		Absence	0	0

*PANO* panoramic radiograph, *LAT* lateral cephalogram, *PA* postero-anterior cephalogram.

Table 4 shows the prevalence of IFs in each 2D radiographic image. Of the 2D images, panoramic radiographs showed the highest prevalence of IFs in all areas when “presence” and “not determined” categories were included as IFs. However, when only definite findings in the “presence” category were considered as IFs, postero-anterior cephalograms showed the highest prevalence of nasal septum deviation. 100 panoramic radiographs and 32 postero-anterior cephalograms were included in the “not determined” group for detecting nasal septum deviation, showing the largest number in the “not determined” categories. However, the proportion of the “not determined” group to the “presence” group was higher in detecting pathologies and maxillary sinus findings. The kappa value provides a simple comparison for distribution of readings. In all categories except nasal cavity, the kappa values were over 0.4, which meant fairly good agreement between the panoramic radiograph readings and CBCT readings. In the soft tissue calcification category, there was a substantial agreement between CBCT readings and lateral cephalogram readings. This applies to the similarity of the distribution and does not guarantee the accuracy of readings, because they can include not only true positive and negative readings, but also false positive and negative readings. Despite this limitation, panoramic radiographs showed more similar distribution than cephalograms, based on the gold standards by CBCT.

Table 5 shows the source of data in the “not determined” category for each 2D radiograph confirmed by the 3D CBCT images. “Not determined” results of panoramic radiographs and postero-anterior cephalograms in the maxillary sinus category, panoramic radiograph in TMJ category, and postero-anterior cephalogram in nasal cavity category were mostly absent in CBCT images. However, all the “not determined” results in soft tissue calcification and pathology categories were present in CBCT images.

### Evaluation of 2D combination radiography diagnostic ability based on the gold standards by CBCT (Table 6)

**Table 6:** Sensitivity, specificity, positive predictive value (PPV), negative predictive value (NPV), and accuracy of 2D combination radiography (PANO + LAT + PA) based on the gold standards by CBCT.

	Assumption 1: “Not determined” as absence					Assumption 2: “Not determined” as presence				
	Sensitivity	Specificity	PPV	NPV	Accuracy	Sensitivity	Specificity	PPV	NPV	Accuracy
Maxillary sinus	65.83	88.33	84.95	72.11	77.08	56.67	93.33	89.47	68.29	75.00
TMJ	55.66	91.51	86.76	67.36	73.58	50.00	95.28	91.38	65.58	72.64
Nasal cavity	76.99	47.44	59.43	67.34	62.22	71.88	58.52	63.41	67.54	65.20
Soft tissue calcification	71.43	100.00	100.00	77.78	85.71	71.43	100.00	100.00	77.78	85.71
Pathology	64.29	100.00	100.00	73.68	82.14	35.71	100.00	100.00	60.87	67.86

Table 6 shows the actual accuracy of 2D radiographs (combination of panoramic radiograph, lateral cephalogram and postero-anterior cephalogram) in detecting IFs compared to 3D CBCT images. We divided the accuracy analysis in terms with two different assumptions: Assumption 1, grouping the “not determined” group as “absence” group; Assumption 2, grouping the “not determined” group as “presence” group. The accuracy of each 2D combination radiograph in detecting IFs is indicated in Table 6, and the highest accuracy was 85.71% observed in the soft tissue calcification findings. However, the lowest accuracy was 62.22% observed in the nasal cavity findings. And in most cases, the accuracy tends to be higher in the case of assumption 1, that is, when it was determined that there is no IFs in the ambiguous case.

## Discussion

Based on the experiences of incidental findings in 3D CT images, several authors have studied their frequency or nature. Although the sample size, mean age, and indication vary, almost all articles showed that over 60% of the samples had incidental findings, from 65.5% to 94.3%. In this study, we detected IFs in 709 (69.5%) of 1020 samples, which is similar to the results of previous studies. Only one article^27^ reported a relatively low prevalence of IFs; only 24.6% of samples had IFs in their 3D CT images. This might be because the range of measurement and the characteristics of the samples were different from those of other studies. Only 18.8% of samples had airway problems in their study, while most of the other studies suggested that they had detected incidental findings of airways in 50% of samples. Lopes et al.^28^ found a similar prevalence rate (18.7%) of incidental findings in airways, but the total prevalence rate (92%) was much higher. The differences came from other abnormalities such as temporomandibular joint (TMJ) lesions, soft tissue calcification, and pathological problems. Given that the definition and range of incidental findings vary depending on the authors, the absolute value of the total prevalence rate might not be meaningful. However, the information of each prevalence rate involves the effectiveness of CBCT in detecting the incidental findings of abnormal structures or lesions.

The originality of this study comes from the fact that large-scale samples of over 1000 subjects were included and the CBCT images were taken with a large FOV. The required size of the FOV varies according to the purpose of the CBCT. In general, small FOVs evaluate limited areas such as the maxillary or mandibular area. Recently, CBCT with a small FOV has been required to evaluate the bone quality of dental implants. Conversely, large FOVs evaluate all craniofacial areas, which include not only the maxilla and mandible but also surrounding areas such as sinuses and airways^18^. Lopes et al. suggested that the frequency of IFs was higher in CBCT images with a larger FOV^29^. In contemporary orthodontics, a large FOV is beneficial for complicated craniofacial or orthognathic cases^30^, and the evaluation of the TMJ area and pharyngeal airway has become more important for orthodontic patients. In addition, CBCT with a large FOV can replace conventional 2D radiographs, such as panoramic radiographs and cephalograms^31^, because the region of interest (ROI) of the orthodontists includes all craniofacial parts, not only the dental area.

Edward et al. suggested that IFs are detected more frequently in older patients than in younger populations^14^. For example, the condylar pathologic changes in patients aged > 65 years were detected 3.6 times more than those in younger patients^32^. Some authors demonstrated that life-threatening pathologies and malignancies were found in CBCT images^18^. Allareddy et al. found three malignancies in 1000 patients^33^, and Warhekar et al. found 11 malignancies in 795 patients^34^. Although the number of subjects in this study was larger than that of previous studies, there were neither malignancies nor life-threatening pathologies found in the CBCT images. The key factor that affects these differences might be the young age of the subjects. As the subjects in this study were patients who visited for orthodontic treatment, they were at a relatively young age of 21.7 years. This is much lower than previous studies whose subjects took CBCT for other purposes such as evaluation for dental implants rather than orthodontic diagnosis: for example, 39.3 years old in Cha et al.’s study^27^, 49.3 years old in Price et al.’s study^35^, and 37.2 years old in Warhekar et al.’s study^34^. Despite the relatively low severity of pathologies found in this study, some of them required therapeutic interventions (Fig. 1). Additionally, we did not find significant difference in age between the subjects with incidental findings and the subjects without incidental findings. A different result in Hernandez et al.’s study showed a significant association between age and the presence of incidental findings^36^. However, they used only panoramic radiographs and lateral cephalograms, a set that showed relatively low accuracy in our study. We used 3D CBCT images and additional postero-anterior cephalograms to achieve a more precise result. Over 80% of our subjects were under 29 years old. Because of the concentrated distribution of age groups, there were no significant differences among age groups.

**Figure 2:**
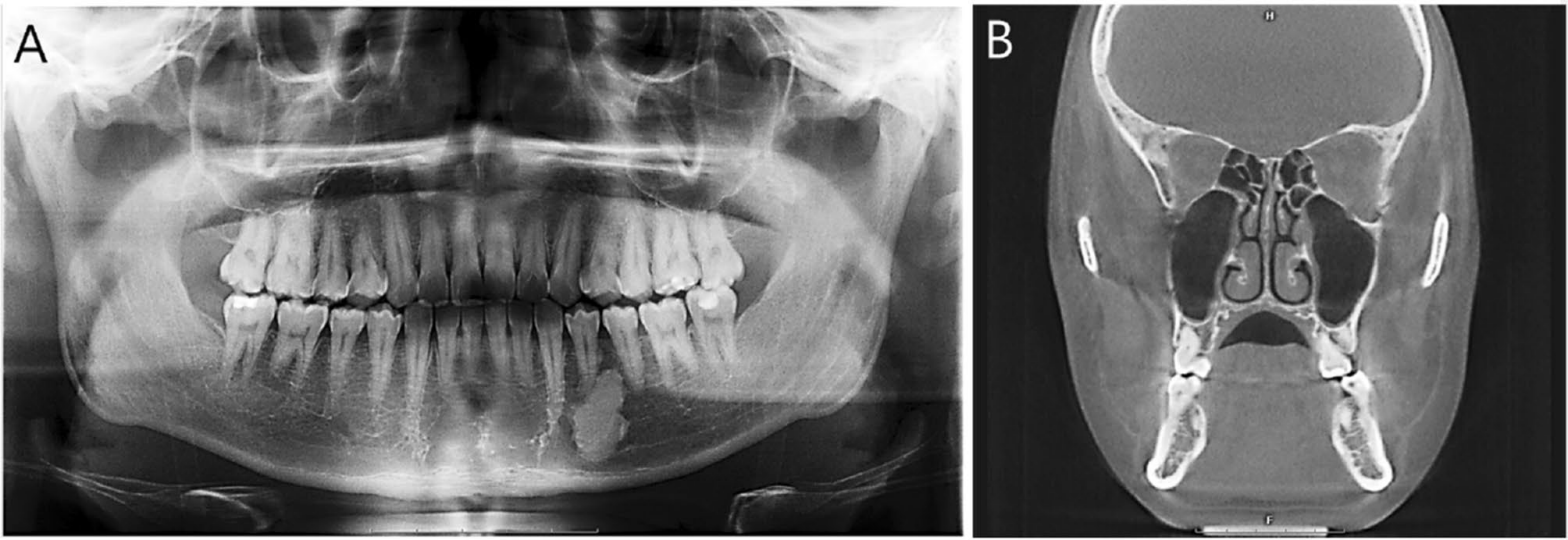
A panoramic image of a 23-year-old male patient seemed to show inflammation of paranasal sinuses based on the haziness of both sinuses, and total opacification was suspected on the right maxillary sinus. However, 3D CBCT image showed clear sinuses without any inflammation. (**A**) Panoramic radiograph, (**B**) clear sinuses in a CBCT slice.

We also focused on the effectiveness of 2D radiographic images. As previous studies investigated the lower accuracy or effectiveness of 2D radiographic images compared to 3D radiographic images, it could be predicted that relatively less false negative findings affect the sensitivity. However, interestingly, there were more false positive findings in 2D radiographic images than we expected. For example, in the case shown in Fig. 2, it seemed to be a haziness of both maxillary sinuses, and a total opacification was suspected on the right sinus, but the sinuses observed in CBCT images were clear without any inflammation.

Unlike with 3D CBCT evaluation, we could not determine the findings in 2D radiographs and they were categorized as “not determined”. In 100 panoramic radiographs of 1020 cases, we could not confirm the nasal cavity findings. As these ratios were higher, the efficiency of 2D radiographs in detecting IFs might be lower. For the conservative interpretation, we regarded the data in the “not determined” category as “absence” of findings when calculating the accuracy. We tried to discover the source of data in the “not determined” category in Table 5. Although the “not determined” data in the soft tissue calcification and pathology categories were all from “presence” of findings in CBCT images, most other data were from “absence” in CBCT results. Hence, we needed the cut-off value accuracy test to confirm the detailed accuracy of each kind of radiograph compared to the gold standard results of the CBCT.

Constantine et al. suggested that panoramic imaging has poor efficiency in finding the problems of maxillary sinuses, presenting a result of 36.7% sensitivity and 51.9% NPV^37^. However, higher sensitivity and NPV of the panoramic radiographs were measured in the present study resulting in higher accuracy. Conversely, the accuracy of lateral and postero-anterior cephalograms was significantly lower than that of the reference, implying a low diagnostic value of cephalograms for detecting maxillary sinus findings. For the assessment of the TMJ area, the specificity was over 90%, but the sensitivity was only about 50% with panoramic radiography. This means that the resorptive changes of condyles could not be examined by panoramic radiographs even in cases with actual resorption of condyles evaluated on CBCT images. This result partially conforms with the explanations by De Grauwe et al., which suggested that CBCT imaging is superior to the conventional 2D radiographic methods for the diagnosis of TMJ abnormalities, and that CBCT can be regarded as a first diagnostic aid instead of 2D radiography^38^. As described above, concha bullosa cannot be found in 2D radiographic images, so only the nasal septum deviation was evaluated in 2D images. The highest accuracy was observed in the postero-anterior cephalogram, and panoramic radiography showed a statistically significant low accuracy. Comprehensive evaluation with the panoramic radiographs and postero-anterior cephalograms also showed a low accuracy, although the difference was statistically insignificant; therefore, accurate findings might be drawn only by the postero-anterior cephalogram without the panoramic radiograph. The specificity and PPV were 100% in all 2D radiographs for the findings of soft tissue calcification and pathology. This resulted in higher accuracy in detecting these abnormalities. Since the subjects who had soft tissue calcification and pathology were relatively small, the reliability of this statistical result might not be secured.

In summary, this study suggests a higher accuracy in detecting the IFs of soft tissue calcification, a moderate accuracy for IFs of the paranasal sinus, TMJ, and pathology, and low accuracy of IFs of the nasal cavity. Maxillary sinus abnormalities on panoramic radiographs were found more accurately than on other 2D radiographs, and nasal septum deviation should be evaluated by postero-anterior cephalograms alone excluding panoramic radiographs when using only 2D radiographic images. Soft tissue calcification can be found with a higher accuracy using comprehensive 2D radiographic images.

When it comes to the radiation issues, there have been controversies about performing CBCT routinely for orthodontic patients, especially in young patients. De Grauwe et al. emphasized that CBCT in a pediatric population can be justified in cases that cannot be diagnosed accurately with conventional radiographs, such as impacted teeth or a cleft lip and/or palate, when the effective dose is higher than the conventional 2D series^38^. The effective dose of CBCT ranges from 11 to 674 μSv (median value, 61 μSv) in small and medium field of view (FOV), and from 30 to 1073 μSv (median value: 87 μSv) in large FOV^39^. The radiation dose of a conventional set of orthodontic radiographs (i.e., panoramic radiograph, and lateral and postero-anterior cephalograms) is 35.81 μSv^40^. That is far less than the effective dose of a multislice CT ranging from 280 to 1410 μSv when providing high-resolution images, used traditionally in medical areas^41^. Based on these advantages, CBCT has been utilized in orthodontics^42^. The ALARA principle, which is an acronym for “as low as (is) reasonably achievable,” has been applied not only in the industrial field but also in the medical field^43^. Despite cautious concerns about radiation exposure, the benefits and risks should be compared. Diagnostic value of CBCT has risen in regular orthodontic patients, not just in specific orthodontic patients, with benefits which could not be achieved by conventional 2D radiographs. Nevertheless, we do not take CBCT images in all regular orthodontic patients for who the conventional 2D radiographic sets have been taken. CBCT images are taken only in patients with special purposes such as the evaluation for orthognathic surgery, impacted teeth, alveolar bone housing, or transverse discrepancy at the furcation level, in cases which had definite benefits. If the benefits were higher than the expected risks, taking required radiographs could be considered to be allowed ethically. Although it is certain that the radiation dose from medical equipment should be minimized, it is known that the risks of being exposed to ionizing radiation used for medical indication are quite low and similar to other risks which are acceptable for everyday life^44^. Moreover, multi factors influence the effective dose for CBCT, and controlling the factors the effective dose of CBCT can be lowered than that of the conventional radiograph set^45^. There is an inevitable drift to use 3D CBCT data in contemporary dentistry, which means the mainstream of imaging in dentistry has become shifted from conventional 2D radiographs to 3D radiographs. This study was meaningful in that the efficiencies of 2D and 3D radiographs to detect IFs were compared in this transition period. With a help of the artificial intelligence technique with deep-learning-based approach, improved quality of CBCT images can be acquired with a lower radiation dose^46^. The detection of IFs may be improved by the development of CBCT equipment with a lower radiation dose and higher resolution.

This study used only radiographic interpretations, using CBCT as the gold standard. A limitation of this study was that there was no histopathological or clinical confirmation of the findings. For example, the findings in a pathology category were expressed based on the radiologic impression, not on the clinical diagnosis. To evaluate the effectiveness of radiographs in detecting IFs, and to overcome the limitation of regarding CBCT images as a gold standard, further studies should match the evaluation of radiographic examinations with clinical information.

The detection of IFs may be improved by the development of CBCT equipment with a lower radiation dose and higher resolution.

In conclusion, (1) 69.5% of subjects showed at least one IFs, so clinicians should be responsible for investigating radiographic images carefully; (2) the possibility of detecting IFs on panoramic, lateral and postero-anterior cephalograms was verified, although the accuracy was not very high; (3) In cases the IFs were not determined because of its ambiguity in 2D radiographs, considering them as an absence of findings increased the accuracy.
